# Rapid estimation of cytosolic ATP concentration from the ciliary beating frequency in the green alga *Chlamydomonas reinhardtii*

**DOI:** 10.1074/jbc.RA120.015263

**Published:** 2020-12-10

**Authors:** Wakako Takano, Toru Hisabori, Ken-ichi Wakabayashi

**Affiliations:** 1 Laboratory for Chemistry and Life Science, Institute of Innovative Research, Tokyo Institute of Technology, Yokohama, Japan; 2 School of Life Science and Technology, Tokyo Institute of Technology, Yokohama, Japan

**Keywords:** algae, ATP, cilia, dynein, photosynthesis, respiration, [ATP], ATP concentration, CBF, ciliary beating frequency, DCMU, 3-(3,4-dichlorophenyl)-1,1-dimethylurea

## Abstract

Determination of cellular ATP levels, a key indicator of metabolic status, is essential for the quantitative analysis of metabolism. The biciliate green alga *Chlamydomonas reinhardtii* is an excellent experimental organism to study ATP production pathways, including photosynthesis and respiration, particularly because it can be cultured either photoautotrophically or heterotrophically. Additionally, its cellular ATP concentration, [ATP], is reflected in the beating of its cilia. However, the methods currently used for quantifying the cellular ATP levels are time consuming or invasive. In this study, we established a rapid method for estimating cytosolic [ATP] from the ciliary beating frequency in *C. reinhardtii*. Using an improved method of motility reactivation in demembranated cell models, we obtained calibration curves for [ATP]–ciliary beating frequency over a physiological range of ATP concentrations. These curves allowed rapid estimation of the cytosolic [ATP] in live wild-type cells to be ∼2.0 mM in the light and ∼1.5 mM in the dark: values comparable to those obtained by other methods. Furthermore, we used this method to assess the effects of genetic mutations or inhibitors of photosynthesis or respiration quantitatively and noninvasively. This sensor-free method is a convenient tool for quickly estimating cytosolic [ATP] and studying the mechanism of ATP production in *C. reinhardtii* or other ciliated organisms.

Measurement of ATP in live cells is important for understanding cellular activities. Various gene-encoded ATP sensors, including the fluorescence-based sensors ATeam (1), B-Queen (2), and MaLionB/G/R (3) and the bioluminescence-based sensor BTeam (4), have been developed for this purpose. These sensors can be expressed in specific cellular compartments, such as the endoplasmic reticulum and mitochondria, and quantitatively monitor local ATP concentrations, [ATP], based on ratiometric analyses. These sensors, however, are not easy to use in some kinds of cells because they must be expressed as recombinant proteins in the target cells, they could perturb the cellular metabolism and the cellular ATP level when overexpressed (5), and in the case of phototrophic organisms, they are susceptible to chloroplast autofluorescence.

In this study, we explored the possibility of easily estimating cytosolic [ATP] in the unicellular green alga *Chlamydomonas reinhardtii* from the beat frequency of its cilia (also called flagella). *C. reinhardtii* is an excellent model organism in various research fields, including photosynthesis, respiration, reproduction, and ciliary function. Because it can be cultured either photoautotrophically or heterotrophically, numerous mutants with defects in photosynthesis or respiration pathways have been isolated (6, 7, 8, 9, 10, 11, 12). Some of these mutant cells swim slower than WT cells (13). Low motility of such mutants may reflect a decrease in their intracellular [ATP].

Eukaryotic cilia are motile organelles driven by microtubule-based motor proteins: dyneins. Ciliary dyneins belong to the protein superfamily containing ATPases associated with diverse cellular activities (AAA+ proteins) and generate force between adjacent doublet microtubules through ATP hydrolysis (14). The inner structure of the cilia, called the axoneme, is detergent-insoluble, and the motility of such a cytoskeleton-based structure has been traditionally studied *in vitro* by detergent extraction followed by the addition of ATP. This kind of experiments is originated from the *in vitro* contraction of glycerinated muscle by Szent-Györgyi (15), and the method was applied for sperm flagella (16), *Paramecium* cilia (17), and then *C. reinhardtii* cilia (18). In each system, after detergent-extraction, motor proteins can be activated by the addition of ATP to show sliding motion against cytoskeletons, and the cell motility can be reproduced *in vitro*. This system enables *in vitro* assessment for the effects of various factors such as ions and nucleotides on cell motility. The detergent-extracted cilia or whole cells (cell models) of *C. reinhardtii* display motility in the presence of ATP such that demembranated cilia beat with almost the same pattern as that in live cells (Fig. 1*A*) (18). The ciliary beat frequency (CBF) of *C. reinhardtii* increases with [ATP] in a Michaelis–Menten pattern (19), as found originally with sea urchin sperm flagella (20). This ATP-dependence of CBF conforming to Michaelis–Menten kinetics is an empirical observation that cannot be theoretically explained as representing the function of single or multiple enzymes. Nevertheless, we have experienced that the [ATP]–CBF curve is reproducible when the same cilia or flagella are tested under constant conditions. We thus hypothesized that cytosolic [ATP] could be estimated by interpolating the CBF of live cells if we can draw a reliable [ATP]–CBF curve from *in vitro* experiments.

**Figure 1:**
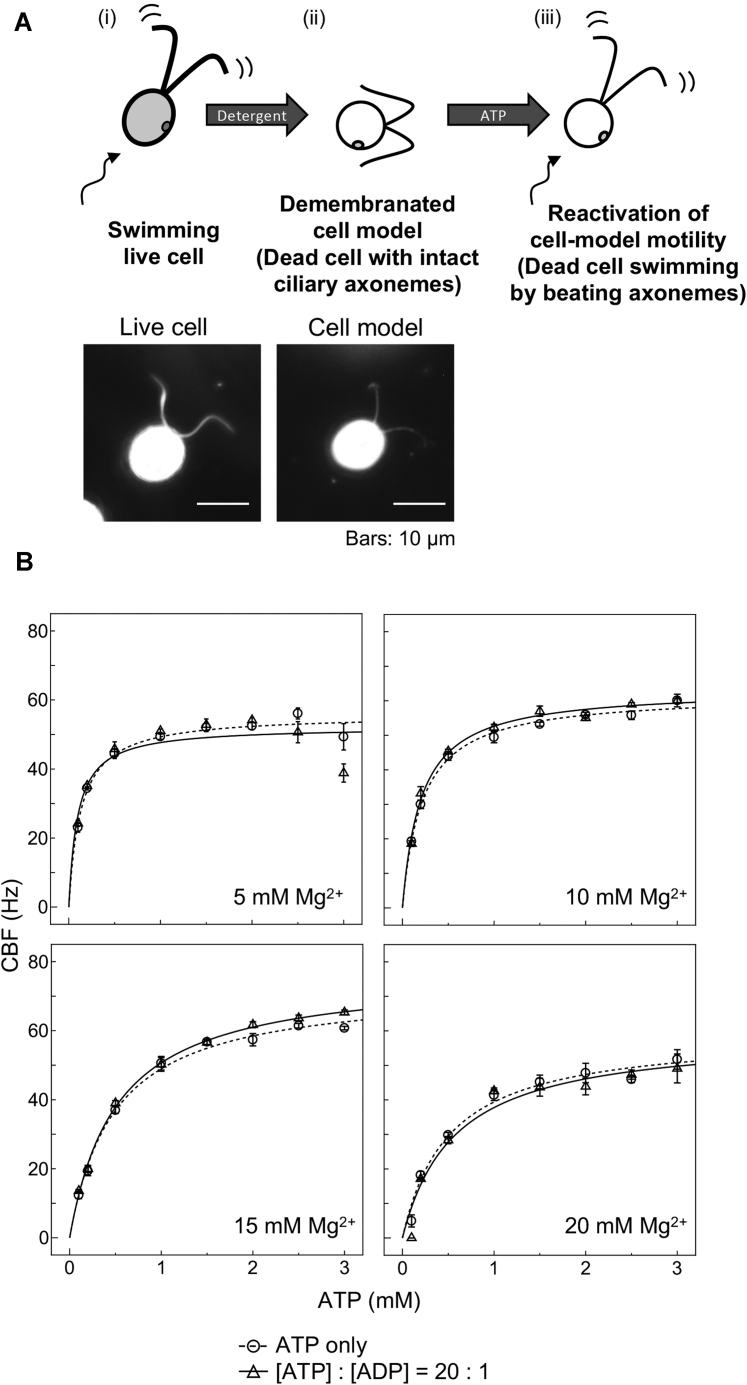
***Chlamydomonas reinhardtii* cell-model system for establishing [ATP]-ciliary beating frequency curves**. *A*, *Top*, schematic images of reactivation of motility in demembranated cell models. Live *C. reinhardtii* cells (i) are treated with a nonionic detergent (final 0.1% Igepal CA-630). Resultant demembranated cell models (ii) are dead and immotile, but the ciliary axonemes are kept intact. The cell bodies are protected by cell walls and do not rupture but become a rounder shape. The addition of ATP (iii) reactivates the beatings of ciliary axonemes. In this study, for the measurement of ciliary beating frequency (CBF), the median frequency was obtained from the power spectra of fast Fourier-transformed vibration signals of cell models in microscope images averaged for ∼20 s. *Bottom*, dark-field micrographs of a live cell (*left*) and a demembranated cell model (*right*) immobilized onto a glass slide. Note that cilia became thinner and the cell body became a rounder shape after demembranation. *B*, [ATP]–CBF curves from reactivated cell models. Motility of WT cell models was reactivated by the addition of ATP with or without ADP under various [Mg^2+^] conditions (the conventional buffer contains 5 mM Mg^2+^). Mean CBFs (±SEM, n = 4) were measured at 0 to 3 mM [ATP]. [ATP]:[ADP] = 20:1 when added. The highest V_max_ (maximal CBF) and the best fitting to the Michaelis–Menten curve were both given under the conditions 15 mM [Mg^2+^] with ADP (see Table 1).

The methods for preparing detergent-extracted cell models of *C. reinhardtii* and reactivating their motility with the addition of ATP are well established and have been used in various studies (13, 18, 21, 22). However, all of these methods are flawed in that the maximal CBF of the cell models under physiological [ATP] is lower than the CBF of live cells. Thus, an improved method has been awaited.

In this study, we improved the conditions for motility reactivation of the cell models so that they display higher CBF comparable to the *in vivo* CBF over a more extensive [ATP] range. Using the improved [ATP]–CBF curve for calibration, we estimated intracellular [ATP] in cells under various conditions. The values thus obtained showed a reasonable agreement with those measured based on luciferin/luciferase phosphorescence. The CBF-based method is entirely noninvasive and rapid, and it is useful for monitoring the dynamics of cellular [ATP] in live cells.

## Results

### Improving conditions for reactivation of cell models

To draw a better [ATP]–CBF curve and estimate the cellular [ATP] from CBF, we first improved the buffer conditions for motility reactivation in detergent-extracted cell models. In previous studies, a reactivation buffer was used that contained 30 mM Hepes (pH 7.4), 5 mM MgSO_4_, 1 mM dithiothreitol, 1 mM EGTA, 50 mM potassium acetate, and 1% polyethylene glycol (Mw: 20,000) (18, 21). The motility of the cell models is readily reactivated by adding ATP to the cell models in this buffer. However, in these conventional experimental conditions, at [ATP] > 1 mM, CBF is lower than projected from the Michaelis–Menten curve (Fig. 1*B*). Because the physiological [ATP] is suggested to be 1 to 2 mM in plant cells (23, 24) and *C. reinhardtii* WT cells swim with a CBF higher than the maximal CBF (V_max_) calculated from Michaelis–Menten kinetics, this problem might be caused by nonoptimal experimental conditions.

Assuming that ATP uncoordinated with Mg^2+^ increases when [ATP] is increased with limited [Mg^2+^] (Fig. S1) and that such free ATP may inhibit dynein ATPase (25), we changed [Mg^2+^] in the buffer to 5 to 20 mM. The CBF, measured from the cell body vibration (26), showed that 15 mM MgSO_4_ conditions yielded a curve well fitted to the Michaelis–Menten equation and gave the highest V_max_ value, which was higher than the CBF in live cells (Table 1). The increase in V_max_ with [Mg^2+^] may mean that Mg^2+^-free ATP indeed inhibits the mechanochemical cycle of dyneins.

**Table 1:** K_m_, V_max,_ and |R| values for Figure 1*B*

Mg^2+^	ATP only			[ATP]:[ADP] = 20:1		
	K_m_ (mM)	V_max_ (Hz)	|R|	K_m_ (mM)	V_max_ (Hz)	|R|
5 mM	0.14 ± 0.044	56.00 ± 3.669	0.9837	0.10 ± 0.016	52.33 ± 2.111	0.8665
10 mM	0.22 ± 0.045	62.11 ± 2.920	0.9963	0.21 ± 0.034	63.47 ± 1.366	0.9945
15 mM	0.49 ± 0.081	72.85 ± 1.156	0.9973	0.52 ± 0.066	76.90 ± 1.640	0.9989
20 mM	0.52 ± 0.010	59.70 ± 5.027	0.9888	0.58 ± 0.108	59.57 ± 4.992	0.9735

As an additional improvement, we added ADP together with ATP. Several studies have suggested that ADP enhances dynein motor activity (27, 28). A previous study showed that in *C. reinhardtii* cells, the ATP: ADP ratio is almost always ∼20:1 (29). We thus added ADP at 1/20 concentration of ATP for reactivation of the motility of the cell models. The addition of ADP resulted in a curve showing an excellent fit to the Michaelis–Menten equation and a higher V_max_ (Fig. 1*B*, Table 1). Based on the excellent *in vitro* motility attained, we decided to use the [ATP]–CBF curve obtained in the buffer containing 15 mM MgSO_4_ and ADP, in addition to other ordinary components, to estimate the cellular ATP concentration from the CBF of live cells (Videos S1–S4).

### Estimation of cytosolic [ATP] from CBF

**Figure 2:**
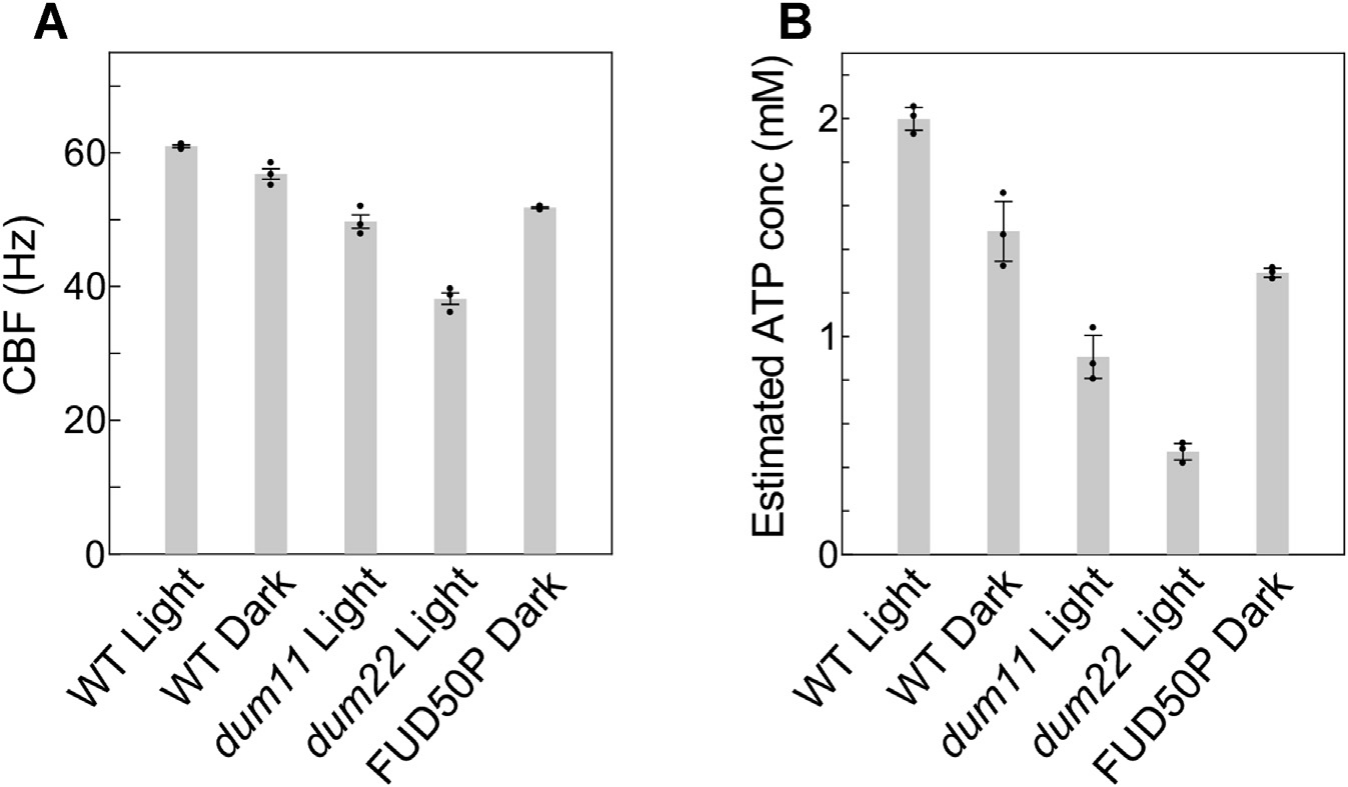
**Measurement of CBF and its conversion to [ATP] in WT and ATP-production mutants.***A*, the CBF of each strain or condition. *B*, the CBF was converted to [ATP] by each strain’s calibration curve (see Fig. S3). Mean values (±SEM, n = 4) are shown as bars and individual data point is plotted. CBF, ciliary beating frequency.

The mean CBF of live WT cells (see Table 2 for the strains used in this study) grown in the light or the dark was measured as 61.0 ± 0.2 or 56.8 ± 0.8 Hz, respectively (mean ± SEM, n = 4) (Fig. 2*A*). These values corresponded with 2.0 ± 0.1 and 1.5 ± 0.1 mM [ATP], respectively, in the 15 mM Mg^2+^ curve of Figure 1*B* (and Fig. S1). It is reasonable that dark-adapted cells, not undergoing photosynthesis, showed lower CBF and cytosolic [ATP] than light-adapted cells.

**Table 2:** Strains used in this study

Strain	Description	Reference
Wild type (WT)	A progeny of the cross CC-124 × CC125, *agg1*^+^, *mt*^−^	This study
CC-124	Wild-type strain, *nit1*^−^, *nit2*^−^, *agg1*^−^, *mt*^−^	(44)
CC-125	Wild-type strain, *nit1*^−^, *nit2*^−^, *mt*^+^	(44)
FUD50	Deletion in the *atpB* gene encoding β subunit of chloroplast F1ATPase in the chloroplast genome. Lacking chloroplast F_o_F_1_ ATPase.	(30)
FUD50P	A progeny of the cross FUD50 (mt^+^) × WT strain (mt^−^)	This study
*dum11*	Deletion of 0.7 kb in the *cob* gene and adjacent end of the mitochondrial genome. Lacking complex III. Respiration deficient.	(31)
*dum22*	Deletion in the cob gene and the 3′ end of the *nd4* gene. Lacking complexes I and III. Respiration deficient.	(32)
*oda1*	Mutation in the ODA1 locus corresponds to the gene encoding DC2 of the outer dynein arm docking complex (ODA-DC). Lacking outer arm dynein and the ODA-DC.	(45)
*ida9*	Mutation in the IDA9 locus corresponds to the gene encoding DHC9, a dynein heavy chain for inner arm dynein subspecies c.	(46)

Next, we assessed the cytosolic [ATP] of several photosynthetic or respiratory mutants listed in Table 2. Before measuring live-cell CBF in these mutants, we examined the motility of their demembranated cells reactivated at different ATP concentrations to verify that the ciliary properties are unchanged. [ATP]–CBF curves (Fig. S3) were used to extrapolate and compare the V_max_ values (Table S1). Mutants used were FUD50P lacking the beta subunit of chloroplast F_o_F_1_ATP synthase (CF1β) as a photosynthesis-deficient mutant (30) and *dum11* and *dum22* as respiratory mutants (31, 32). Because the original FUD50 cilia contain less of an outer-arm dynein component probably caused by unintentional mutation, FUD50P, a progeny of the cross WT × FUD50 was used (see Experimental procedures and Fig. S2). The *dum11* strain lacks complex III, and the *dum22* strain lacks complexes I and III in the respiration chain in mitochondria. The cytosolic [ATP] of these stains were calculated from each strain’s [ATP]–CBF curve (Fig. S3).

The cytosolic [ATP] for each mutant was calculated from the respective CBF values of live mutant cells (Fig. 2). Because FUD50P does not grow in the light and *dum11* and *dum22* do not grow in the dark, these strains were kept only in their preferred dark or light conditions, respectively. The cytosolic [ATP] of FUD50P was 1.3 ± 0.1 mM, which was close to the value of dark-grown WT (1.5 ± 0.1 mM). Consistently, dark-grown WT and the photosynthesis-deficient mutant showed similar cytosolic [ATP] values (Fig. 2). By contrast, the respiration mutants showed significantly lower [ATP] values (0.9 ± 0.1 mM in *dum11* and 0.5 ± 0.1 mM in *dum22*) than the other strains, suggesting that respiration contributes to the cytosolic [ATP] more significantly than does photosynthesis under heterotrophic culture conditions (Fig. 2).

For a more straightforward estimation of the cytosolic [ATP] in a mutant, its CBF can be extrapolated into the WT calibration curve instead of its own. The [ATP] values estimated from the WT calibration curve were not significantly different from those from the respective strain’s curve, except for FUD50P (Table 3). This difference may be caused by the remaining unintentional mutations in FUD50P, suggested from a slightly lower V_max_ value of the calibration curve (Table S1, Fig. S3). Therefore, when estimating the cytosolic [ATP] from CBF, the calibration curve should be changed depending on the purpose: the respective strain’s calibration curve for better estimation and the WT calibration curve ([ATP] =0.52 mM ∗CBF/(76.9 Hz-CBF)) for easier estimation. We employed the former in this study below.

**Table 3:** Comparison of [ATP] estimated by different calibration curves

Strain and light condition	Estimated [ATP] (mM)		*p* value^a^
	By WT calibration curve	By each strain's calibration curve	
*dum11*, light	0.96 ± 0.096	0.91 ± 0.098	0.3120
*dum22*, light	0.51 ± 0.039	0.47 ± 0.038	0.1700
FUD50P, dark	1.07 ± 0.015	1.29 ± 0.021	0.0001

a Student’s *t* test.

### Estimation of cellular [ATP] by the bioluminescence-based method

To validate the [ATP] values estimated from CBF, we next measured the intracellular ATP concentration by a bioluminescence-based method. Whole-cell extracts were prepared from each strain after TCA fixation, and the ATP amounts in cell lysates were measured by an ATP detection system based on the luciferin-luciferase reaction. To convert the ATP amount to its intracellular concentration, we approximated cells to spheres and calculated the total cell volume from the cell number and the mean cell radius (Fig. S4).

**Figure 3:**
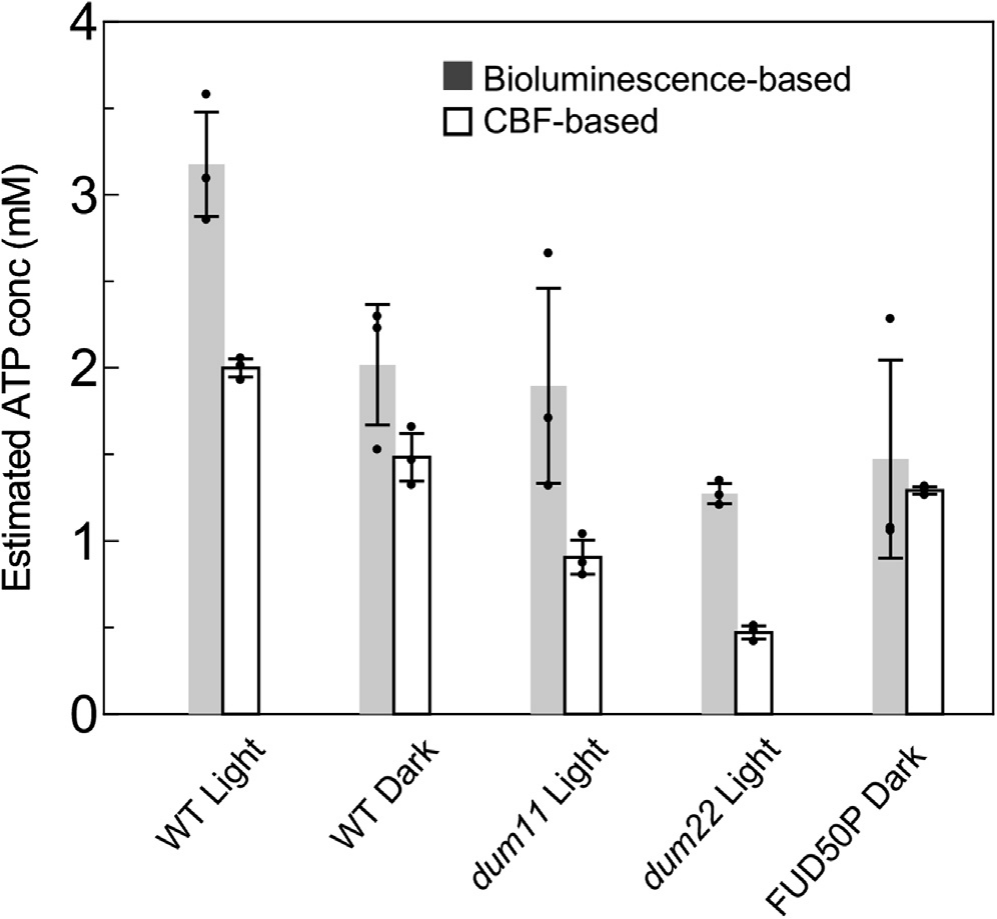
**Cellular [ATP] concentrations estimated by the bioluminescence-based (*gray*) or by the CBF-based (*white*) method.** For both methods, [ATP] was measured using a culture in the early log phase. For the bioluminescence-based method, the cell number and cell diameters in the culture were measured, and the ATP amount in the cell lysates was converted to a function of the cell volume. CBF was measured using the same culture 1 h before the cell lysate preparation for the bioluminescence-based method. Mean values (±SEM, n = 4) are shown as bars, and individual data point is plotted. CBF, ciliary beating frequency.

The bioluminescence-based method yielded significantly higher [ATP] values than those estimated by the CBF method. However, both methods reported the same patterns in the [ATP] difference among cells under different physiological or genetic conditions (Fig. 3). The difference in the [ATP] values assessed by the two methods may not be surprising because the CBF reflects the [ATP] in cilia, whereas the bioluminescence method measures the total quantity of [ATP] contained in both the cytoplasm and chloroplasts. If we consider that the chloroplast occupies ∼51% of the total cell volume (33), whereas the cytoplasm occupies ∼40% (34), we may consider that [ATP] measured by the bioluminescence-based assay is greatly affected by the [ATP] in the chloroplasts.

### Estimation of the effects of inhibitors on ATP production

**Figure 4:**
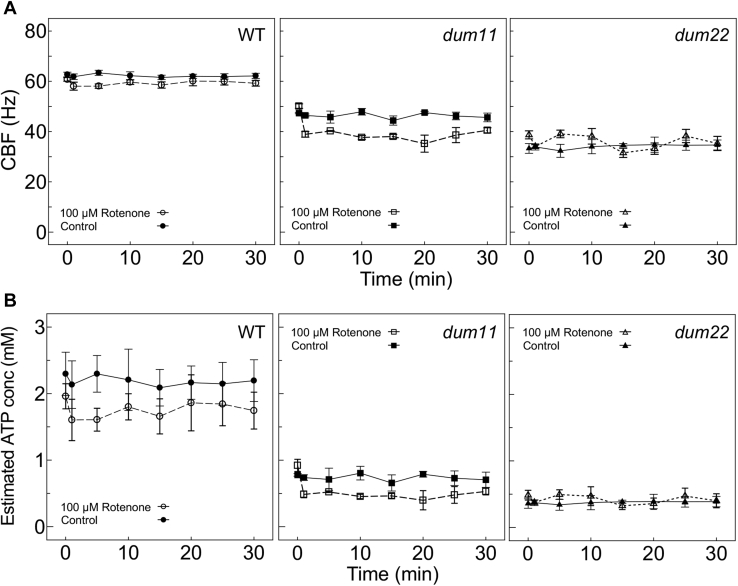
**Effects of rotenone, an inhibitor of the mitochondrial electron transport chain, on the cytosolic [ATP].***A*, 100 μM rotenone or ethanol (control) was added to the culture, and the CBF was measured for 30 min in each strain. *B*, The CBF values were converted to [ATP] using the curves in Fig. S3. Rotenone reduced the CBF in WT and *dum11*, strains with functional complex I, and did not reduce that in *dum22*, a mutant lacking complexes I and III. CBF, ciliary beating frequency.

Taking advantage of the rapidity of the CBF-based method, we assessed the effects of two kinds of inhibitors on ATP production. First, we tested rotenone, a respiration inhibitor that targets complex I. WT, *dum11*, and *dum22* cells were treated with 100 μM rotenone, and the CBF of each strain was measured for 30 min. The CBFs of *dum11* and *dum22* were lower than those of the WT before rotenone treatment (Fig. 4*A*). After the treatment, the CBFs of WT and *dum11* (lacking complex III) cells decreased within 1 min, whereas the CBF of *dum22* (lacking complexes I and III) cells assumed a low level at time zero that did not decrease further with time (Fig. 4*A*). These data suggest that rotenone inhibited complex I in the respiratory chain. These changes in CBF were converted to the change in the cytosolic [ATP] from each strain’s calibration curve (Fig. S3). Rotenone decreased cytosolic [ATP] in WT cells from 2.0 ± 0.2 to 1.6 ± 0.3 mM within 1 min (Fig. 4*B*), suggesting that respiration contributes to the cytosolic [ATP] by ∼0.4 mM under normal conditions.

**Figure 5:**
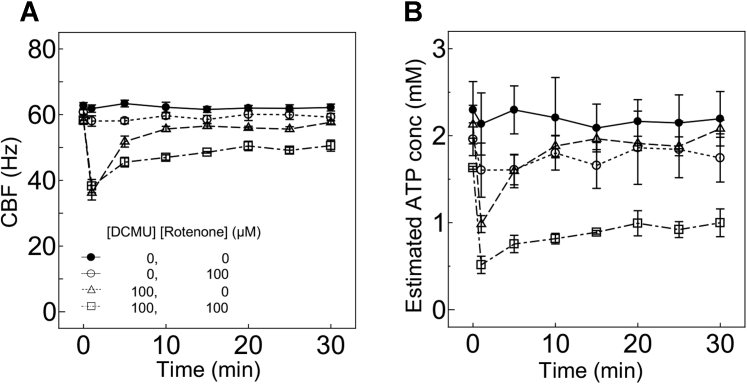
**Effects of DCMU, an inhibitor for the photosynthetic electron transport chain, on the cytosolic [ATP].***A*, 100 μM DCMU, 100 μM rotenone, or ethanol was added to the WT culture, and the CBF was measured for 30 min. *B*, the CBF values were converted to [ATP]. The CBF/cytosolic [ATP] was reduced within 1 min after the addition of 100 μM DCMU and then gradually recovered. This recovery was suppressed by the simultaneous addition of rotenone. CBF, ciliary beating frequency; DCMU, 3-(3,4-dichlorophenyl)-1,1-dimethylurea.

Next, we tested 3-(3,4-dichlorophenyl)-1,1-dimethylurea (DCMU), a photosynthesis inhibitor that targets photosystem II. CBF decreased within 1 min after treatment with DCMU and recovered almost completely by 30 min (Fig. 5*A*). Conversion of CBF to [ATP] showed that, similarly, the cytosolic [ATP] dropped from 2.1 ± 0.2 to 1.0 ± 0.1 mM immediately after the DCMU treatment and spontaneously recovered to 2.1 ± 0.1 mM within 30 min (Fig. 5*B*). These data suggest that photosynthesis contributes to cytosolic [ATP] by ∼1.1 mM under heterotrophic culture conditions. We surmised that the activation of respiration is primarily responsible for this recovery. To test this idea, we treated the cells with both DCMU and rotenone. After the treatment, [ATP] decreased from 1.6 ± 0.1 to 0.5 ± 0.1 mM within 1 min and partially recovered to 1.0 ± 0.2 mM by 30 min (Fig. 5*B*). This modest recovery supports the concept that respiration activation is indeed crucial for recovery but simultaneously suggests that other metabolic pathways, such as glycolysis, also play a role.

## Discussion

We estimated the intracellular [ATP] in *C. reinhardtii* from CBF *in vivo*, using *in vitro* data on the [ATP]-dependence of ciliary movement in demembranated cells (cell models). Measurements of CBF in several metabolic mutants or cells treated with metabolic inhibitors allowed us to quantify the contribution of photosynthesis and respiration to the cytosolic [ATP].

### Validity of measuring cytosolic [ATP] from CBF

CBF must reflect [ATP] in the cilia, but whether or how much this level differs from the cytosolic [ATP] is unclear. Because ATP molecules are produced in the cell body and diffuse into the cilia, where they are consumed by axonemal dyneins and other ATPases such as intraflagellar transport motors, the ATP concentration along each cilium could form a steep gradient (35). However, studies have shown that molecules as small as ATP can quickly diffuse from the base to the tip of the sperm tail (36, 37) and that glycolytic enzymes and adenylate kinase present in cilia may compensate the ATP level (38, 39). Thus, the ATP concentration could be almost homogeneous along the ciliary length, and the average concentration may be close to, or slightly lower than, the cytosolic [ATP]. We thus assume that intraciliary and intracellular ATP levels are comparable to each other and vary in the same manner.

The CBF-based [ATP] assessment has an obvious limitation that it is based on the assumption that the ATP-dependence of CBF is the same *in vivo* and *in vitro*. This assumption is not warranted, however, because we do not understand the exact solution conditions within the cilia. Additionally, our assumption does not account for several conditions such as cilia in live cells; that the axonemes in cell models should experience different hydrodynamic loads; and that the CBF at a constant [ATP] could be affected by trace amounts of reactive oxygen species, cAMP, or Ca^2+^ (13, 40, 41). Nevertheless, we believe that the [ATP]–CBF curve we obtained in our improved system provides a sound reference table for estimating [ATP] from CBF, particularly because the CBF in cell models varies over a wide range of ATP concentrations up to 3 mM, at which it is slightly higher than the maximal CBF observed in live cells.

The [ATP]–CBF curve covering the whole range of CBF observed *in vivo* became available by two modifications made to the previous reactivation buffer; in the previous buffer, CBF decreased at physiological [ATP] concentrations of 2 to 3 mM and never reached the maximal CBF observed *in vivo* (Fig. 1*B*). One modification increased [Mg^2+^] from 5 to 15 mM, which resulted in a higher V_max_ value in the [ATP]–CBF curve (Fig. 1*B*, Table 1). The other modification is the addition of a small amount of ADP (1/20 of [ATP]) together with ATP for reactivation, which also increased the V_max_ value in the [ATP]–CBF curve (Fig. 1*B*, Table 1) (29). Although 15 mM seems to be extremely high for cytoplasmic [Mg^2+^], it may not be entirely impossible for a special compartment of the cilia. Likewise, the presence of a small amount of ADP may be possible, as indicated by a previous study (29). Nevertheless, these modifications require validation in future studies.

### Comparison of [ATP] estimated by our method and the bioluminescence method

We measured cellular [ATP] by a widely used method based on the luciferin–luciferase reaction and estimated it from the [ATP]–CBF curve and CBF of live cells. Although the patterns of differences in concentration values attained under different culture conditions or from mutants were consistent with those attained by the bioluminescence-based method, the bioluminescence-based method always produced higher values than those of the CBF-based method. This may be because of a difference in [ATP] between the cellular and ciliary cytoplasm or between the cytoplasm and chloroplasts.

If we consider the latter possibility, it is interesting to note that WT cells and the photosynthesis-deficient mutant FUD50P in the dark showed similar [ATP] measured by these two methods (Fig. 3). Considering that chloroplasts occupy ∼51% of the total cell volume and the cytosol occupies ∼40% (33, 34), we assume that the chloroplast contains almost the same amount of ATP as the cytoplasm under these conditions. In *Arabidopsis thaliana*, the transport of ATP between the cytosol and the chloroplasts is limited, but ATP import from the cytosol to the chloroplast occurs in young seedlings in the dark (24). Such ATP import to the chloroplast may occur in *C. reinhardtii* under nonphotosynthetic conditions.

Theoretically, WT and FUD50P cells in the dark should show the same [ATP] values, because both of them are nonphotosynthetic. However, the values in WT cells seemed slightly higher than those in FUD50P, and the difference between the values estimated by the two methods was larger in WT cells (Fig. 3). These discrepancies can be attributed to technical limitations; both methods cannot be carried out in the complete dark. For the CBF-based analysis, cells were observed with dim red light under a microscope. For the bioluminescence assay, cells were shortly exposed to the room light before the fixation. Methods to estimate cellular [ATP] in the dark should be further considered.

### Contribution of respiration and photosynthesis to the cytosolic [ATP]

The obvious merit of the CBF-based method compared with other methods is its rapidity; once the [ATP]–CBF curve is established *in vitro*, each measurement takes only ∼20 s. Therefore, the CBF-based method allows us to monitor the dynamics of cellular [ATP], unlike an end-point assay based on bioluminescence.

As a proof-of-concept demonstration for this, we assessed the contribution of photosynthesis and respiration to cytosolic [ATP]. Rotenone and DCMU are inhibitors of respiration and photosynthesis, respectively, but their effect on ATP has been analyzed only rarely. Our observations clearly showed that these inhibitors decreased the cytosolic [ATP] of WT cells within 1 min. Taking advantage of the rapidity of the CBF measurement, we found that a decrease in cytosolic [ATP] caused by DCMU could be compensated in ∼10 min by respiration under heterotrophic culture conditions. We hope that this semi-real-time method will be useful to understanding the kinetics of cellular ATP metabolism as it can be applied to any kind of metabolic inhibitors or changes in culture conditions, such as the conversion between photoautotrophic and heterotrophic conditions.

### Another application: mutant screening

Another application of this CBF-based [ATP] estimation could be mutant screening. To screen for the mutants with defects in ATP production pathways, one would use the growth rate for a criterion. However, such mutants may have defects in cell volume as *dum11* and *dum22* in this study (Fig. S4), leading to errors in cell counting or assumptions on the cell volume. The CBF-based [ATP] estimation can be used to screen for slow-swimming mutants. If those slow-swimming mutants show high V_max_ values of CBF *in vitro*, the mutants may have some defects in ATP production pathways.

In conclusion, we developed a method to estimate cytosolic [ATP] from CBF. This method is simple, rapid, and sensor-free; simple microscopic observation for ∼20 s produces an average CBF of *C. reinhardtii* cells, and this CBF can be easily converted to [ATP] in the physiological range. Furthermore, this method can be applied to any ciliated organisms. Although the validity of the absolute [ATP] values estimated from CBF should be confirmed, this method will provide a quantitative understanding of factors that modulate ATP production pathways in *C. reinhardtii* and other ciliated organisms.

## Experimental procedures

### Cell culture and strains

Cells were grown in tris-acetate-phosphate medium with aeration at 25 °C on a 12 h light/dark cycle (light conditions: 50 μmol photons m^−2^ s^−1^, white light) (42). Strains used in this study are listed in Table 2. FUD50 displayed a reduced amount of ciliary outer-arm dynein component (IC2), presumably because of an unintentional mutation (Fig. S2). We thus removed this deficiency of FUD50 by crossing it with WT. The resultant progeny with defects in the chloroplast F1ATPase β subunit and retaining a normal amount of IC2 was used and designated as FUD50P. The photosynthesis mutants were grown in the dark.

### Measurement of ciliary beating frequency

CBF was measured based on a previously described method (26) with modifications (13). In brief, a photodetector was set on the top of a microscope equipped with a dark-field condenser (BX-53; Olympus). Cells were observed under the microscope with dim red light (λ > 630 nm) to avoid the accumulation of cells caused by phototaxis. Signals derived from cell body vibration were detected by the photodetector, transferred to the computer soundboard, and fast-Fourier transformed using SIGVIEW (SignalLab). Transformed signals were averaged for ∼20 s. The median frequency was regarded as CBF. The resultant [ATP]–CBF plot was fitted to the Michaelis–Menten equation by Ngraph (http://www2e.biglobe.ne.jp/∼isizaka/indexe.htm).

### Micrographs

Cells or cell models were immobilized onto a glass slide coated with 0.1% polyethyleneimine, observed under a microscope equipped with a dark-field condenser (BX-53; Olympus) and photographed by using a CMOS camera (STC-5MUSB3; Sentech).

### Reactivation of demembranated cell models

Cell models were prepared and analyzed using a previously described method (18) with modifications. The regular reactivation buffer contains 30 mM Hepes, pH 7.4, 5 mM MgSO_4_, 1 mM dithiothreitol, 1 mM EGTA, 50 mM potassium acetate, and 1% polyethyleneglycol (Mw: 20,000). To this buffer, MgSO_4_ was added to final concentrations of 10, 15, or 20 mM. cOmplete, EDTA-free Protease Inhibitor Cocktail Tablets (COEDTAF-RO; Roche) were added to the reactivation buffer. For reactivation of motility, ATP was added to the cell models with or without ADP at the ratio [ATP]:[ADP] = 20:1.

### Estimation of free ATP

The concentration of free ATP, which is not coordinated with Mg^2+^, was calculated using the program CALCON (http://www.bio.chuo-u.ac.jp/nano/calcon.html).

### Measurement of intracellular ATP concentration using bioluminescence

Intracellular ATP concentrations were measured by the commercially available bioluminescence-based kit (ATP Bioluminescence Assay Kit CLS II, 11699695001; Roche). Cells were fixed with a final 1% TCA on the same day of the CBF measurement when the culture density reached 1 × 10^6^ cells/ml. Fixed cells were subjected to ATP measurement following the manufacturer’s instructions in the kit and a luminometer (Model BLR-201; Aloka). For conversion of the ATP amount in the cell lysate to the cellular ATP concentrations, total cell volume was calculated from the cell number, and the single-cell volume was estimated by approximating cells as spheres. The cell number and the average cell diameter were measured by an automatic cell counter (model R1; Olympus).

### Treatment with metabolic inhibitors

After harvesting by centrifugation, cells were suspended in fresh tris-acetate-phosphate medium at 5 × 10^6^ cells/ml and placed under white light (50 μmol photons m^−2^ s^−1^) for 30 min. Rotenone or DCMU was added to the cell suspension at a final concentration of 100 μM, and CBF was measured for 30 min. The 0 min time point was analyzed immediately before the addition of the inhibitors.

### Western blotting

Cilia were isolated by a previously reported method (43), demembranated with 0.1% Igepal-CA630 (I3021; Sigma-Aldrich), and subjected to Western blotting. The anti-ODA-IC2 antibody (D6168; Sigma Aldrich) was used as a primary antibody, and anti-mouse IgG (NA931; GE Health Care) was used as a secondary antibody.

### PCR for detection of FUD50 mutation

For detection of the deletion in the FUD50 gene, the following primers were used: 5′-GTGGATTTACATGGCCAGGTGATTATTTAC-3′ and 5′-CGATGCTCGATCTTGGTTTTTCTTAATTAG-3′.

## Data availability

All data are contained within the manuscript.

## Conflict of interest

The authors declare that they have no conflicts of interest with the contents of article.
