# Supplementary material for: Rapid estimation of cytosolic ATP concentration from the ciliary beating frequency in the green alga Chlamydomonas reinhardtii
Source: J Biol Chem. 2020 Dec 10;296:100156. doi: 10.1074/jbc.RA120.015263 (PMC7857514; doi:10.1074/jbc.RA120.015263)
Supplement: Video S4 [file mmc5.pdf]

Supplemental Information

Table S1. K_m, V_{max}, and |R| values for Fig. S3.

	15 mM Mg^{2+}, [ATP] : [ADP] = 20 : 1		
strain	K_m (mM)	V_{max} (Hz)	|R|
WT	0.52±0.066	76.90±1.640	0.9989
dum11	0.4375±0.0267	73.93±0.679	0.9981
dum22	0.4239±0.0264	72.56±0.380	0.9922
FUD50P	0.5066±0.0370	71.11±2.860	0.9972
oda1	0.34±0.127	32.58±3.057	0.9987
ida9	0.33±0.117	60.64±3.164	0.9941

Fig. S1

Estimation of Mg^{2+}-free ATP in the buffer for motility reactivation at various ATP concentrations with (left) or without (right) ADP. The reactivation buffer contains following compounds: 30 mM Hepes, pH 7.4, 1 mM dithiothreitol, 1 mM EGTA, 50 mM potassium acetate, 1% polyethyleneglycol (Mw: 20,000). To this buffer, $MgSO_4$ was added to final concentrations of 5, 10, 15, or 20 mM. The concentration of Mg^{2+}-free ATP, which inhibits axonemal motility, varies in the buffers containing different Mg^{2+}, ATP, and ADP concentrations. The 5 mM Mg^{2+} conditions provide significantly higher [Mg^{2+}-free ATP] than the other conditions in the range 1 ≤ [added ATP] ≤ 3 mM, which would be the main cause of low CBF of reactivated cell models in these [ATP] conditions.

Fig. S2

(A) Cross of FUD50 (mating type plus) ×WT (mating type minus). Progenies A1 and A2 were subjected to subsequent analyses. Note that the chloroplast genome inherits from the mating-type-plus parent to all four progenies. (B) Schematics to show the positions of primers to check deletion in the *ATPB* gene (encoding CF1β) in the chloroplast genome. (C) PCR analysis to check the deletion in the *ATPB* gene. Both A1 and A2 showed deletion in ATPB. A1 was used as FUD50P. (D) CBF in FUD50 was lower than that in WT, and this was recovered by back-cross with WT. Mean values ± SEM are shown (n = 3) as bars and individual data point is plotted. (E) Western blotting of cilia samples using the anti-IC2 antibody (top) and CBB stained tubulin bands of the same sample (bottom). (F) The relative amount of IC2 in cilia normalized by the amount of tubulin (WT = 1). Mean values ± SD are shown (n = 3) as bars and

individual data point is plotted. FUD50 cilia contained a reduced amount of IC2 compared with that of WT cilia, which was recovered by back-cross with WT. IC2, intermediate chain 2 in outer-arm dynein;

Fig. S3

[ATP]-CBF curves for the strains listed in Table 2. Each curve is drawn from the reactivation experiment of motility of the demembranated cell models prepared from each strain. Mean values (±SEM, n = 4) are plotted and fit with the Michaelis–Menten curve. The reactivation buffer contained 15 mM Mg^{2+} and ADP at 1/20 molar ratio of ATP. The dynein-deficient strains *oda1* (lacking outer-arm dyneins and the ODA-DC) and *ida9* (lacking inner-arm dynein c) were used as the controls for low V_{max} values (see Table S1). CBF, ciliary beating frequency.

Fig. S4

Cell density, CBF, cell diameter, and cell volume during the culture. CBF measurement and the cell lysate preparation were performed when the cell density reached 1×10^6 cells/mL. Total cell volume was calculated from the cell density with the cell volume approximating a sphere. CBF, ciliary beating frequency.

Video S1

WT cell models reactivated with 0.2 mM ATP + 0.01 mM ADP in a buffer containing 15 mM Mg^{2+}.

Video S2

WT cell models reactivated with 0.5 mM ATP + 0.025 mM ADP in a buffer containing 15 mM Mg^{2+}.

Video S3

WT cell models reactivated with 1.5 mM ATP + 0.075 mM ADP in a buffer containing 15 mM Mg^{2+}.

Video S4

WT cell models reactivated with 2.0 mM ATP + 0.1 mM ADP in a buffer containing 15 mM Mg^{2+}.

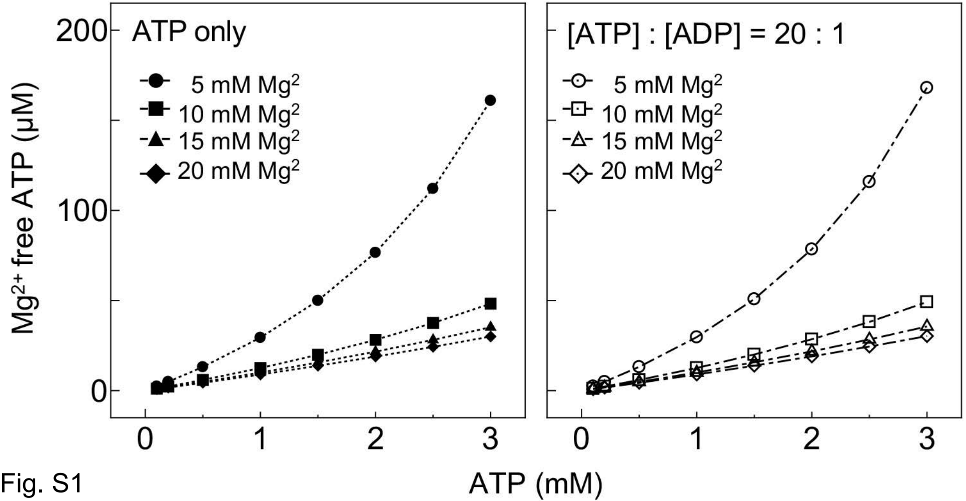
ATP only
5 mM Mg2
10 mM Mg2
15 mM Mg2
20 mM Mg2
[ATP] : [ADP] = 20 : 1
5 mM Mg2
10 mM Mg2
15 mM Mg2
20 mM Mg2
Mg2+ free ATP (μM)
200
100
0
0
1
2
3
0
1
2
3
ATP (mM)

Fig. S1

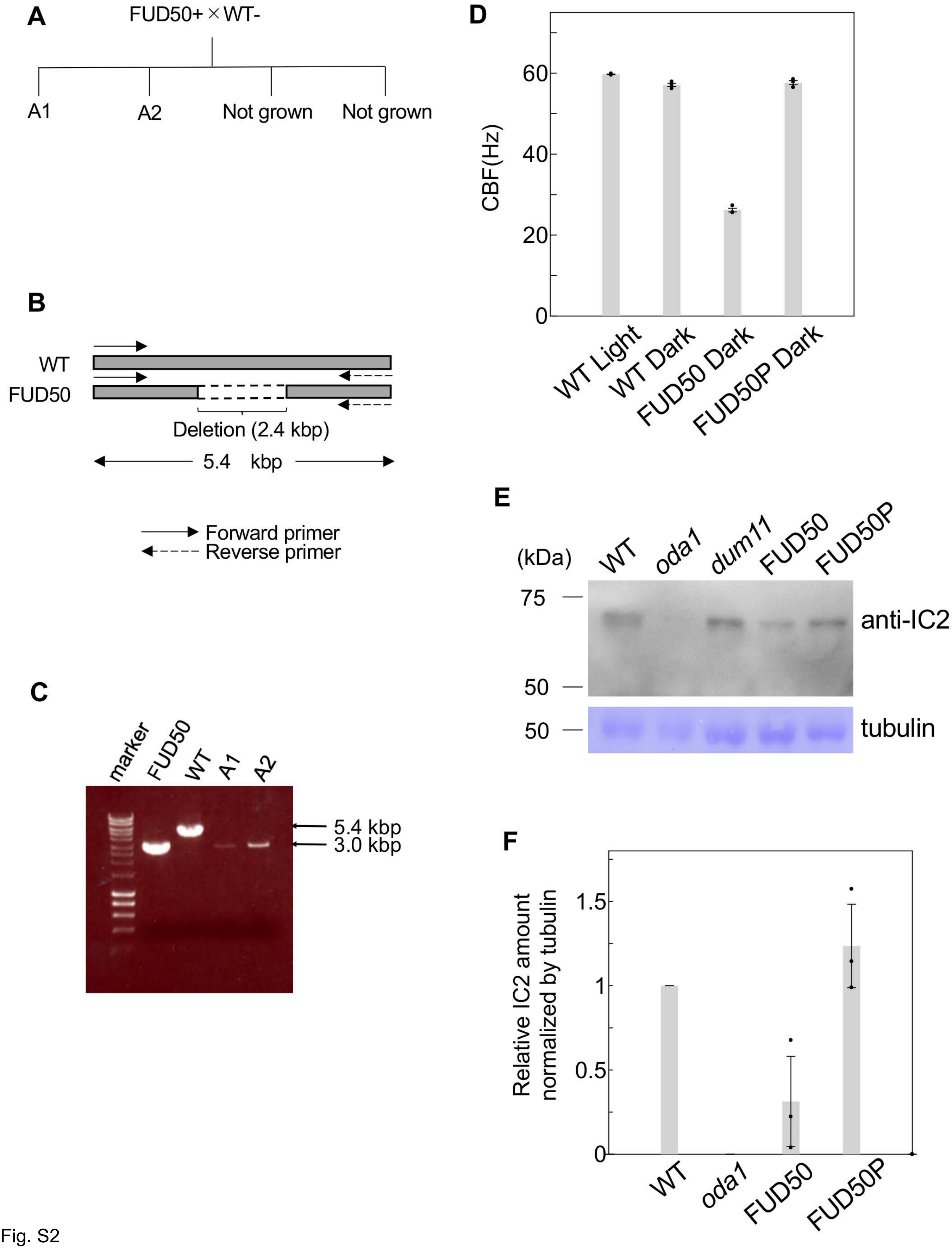
A
FUD50+ × WT-
A1
A2
Not grown
Not grown
B
WT
FUD50
Deletion (2.4 kbp)
5.4 kbp
Forward primer
Reverse primer
C
marker
FUD50
WT
A1
A2
5.4 kbp
3.0 kbp
D
CBF(Hz)
60
40
20
0
WT Light
WT Dark
FUD50 Dark
FUD50P Dark
E
(kDa)
WT
oda1
dum11
FUD50
FUD50P
75
50
anti-IC2
50
tubulin
F
Relative IC2 amount
normalized by tubulin
1.5
1
0.5
0
WT
oda1
FUD50
FUD50P

Fig. S2

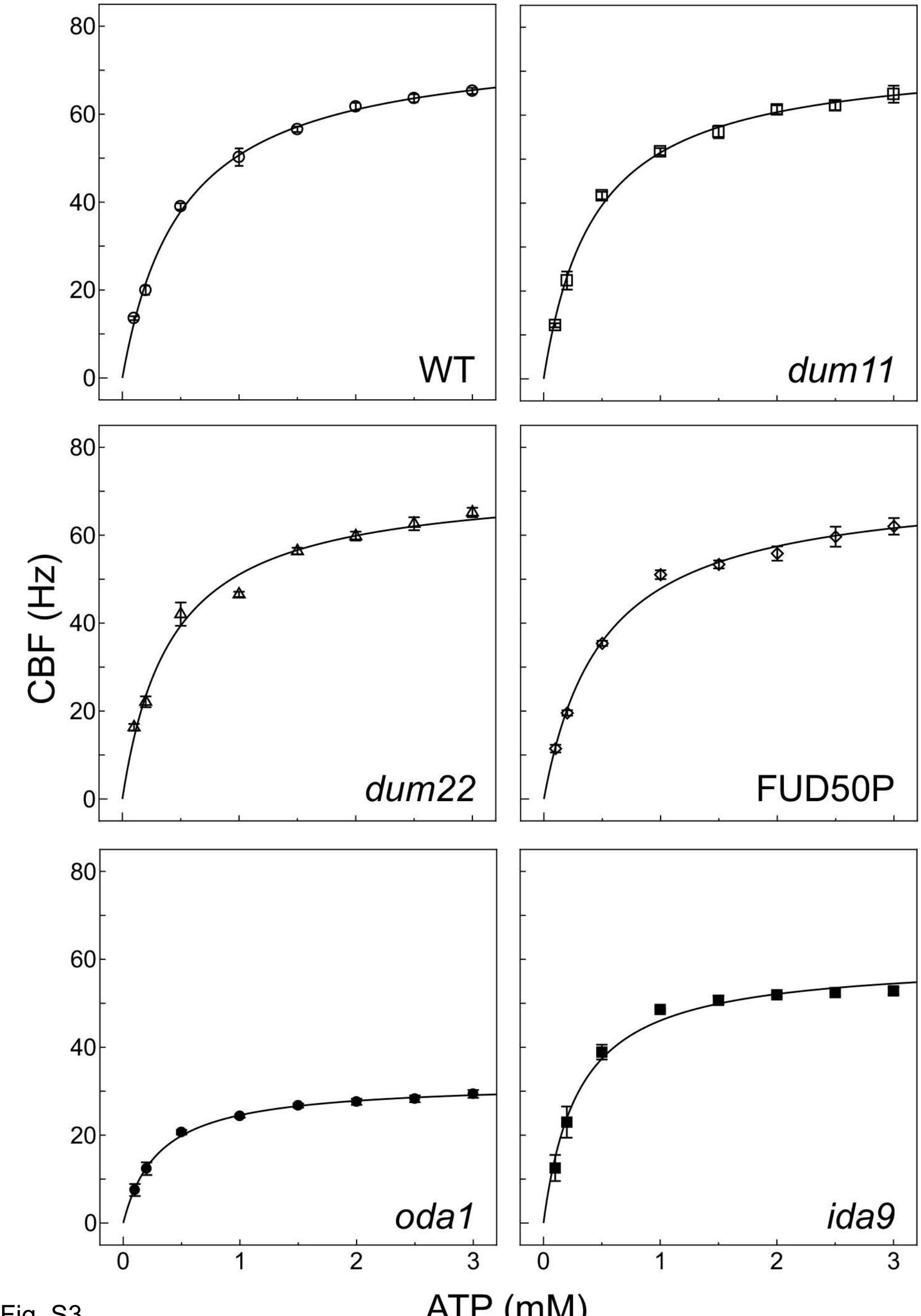

Fig. S3

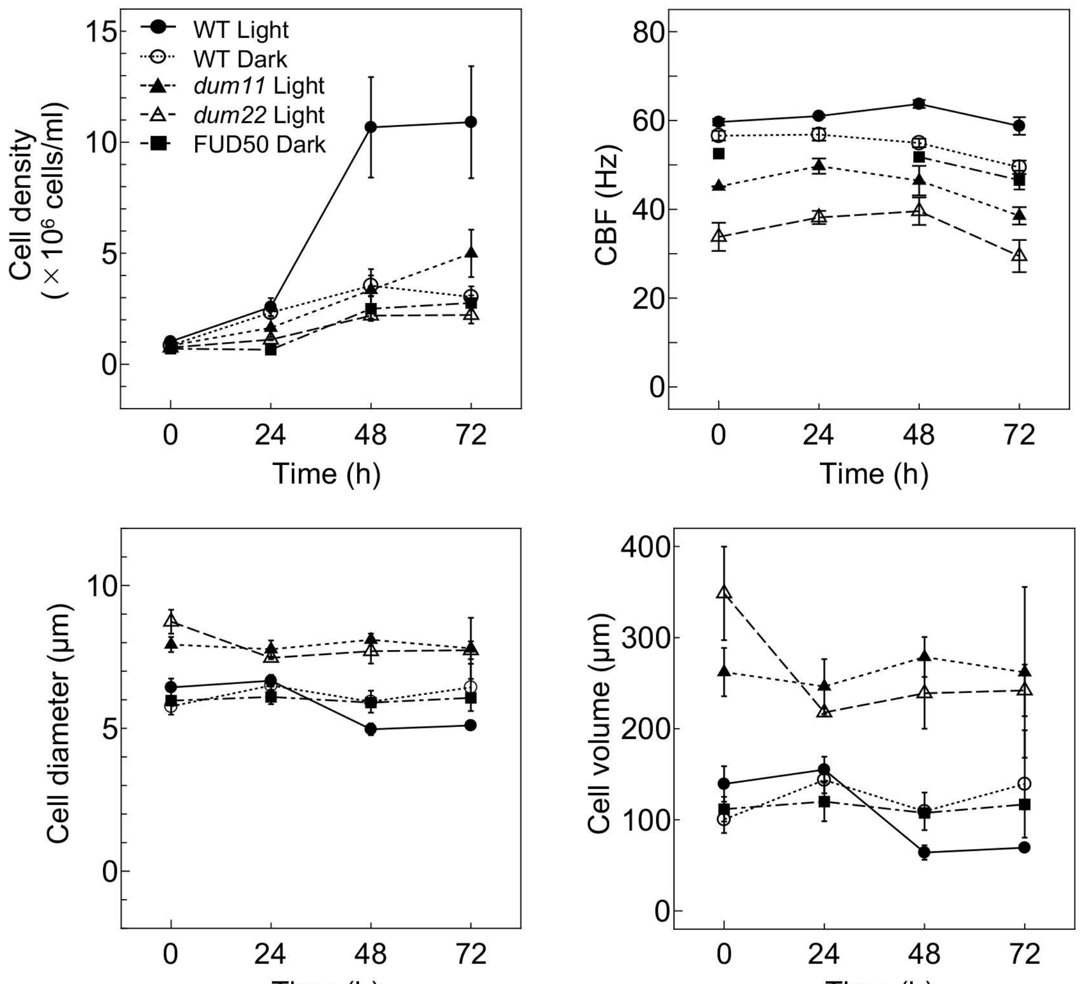

WT Light
WT Dark
dum11 Light
dum22 Light
FUD50 Dark
Cell density
(× 10[6] cells/ml)
Time (h)
CBF (Hz)
Cell diameter (µm)
Cell volume (µm)
0
24
48
72

Fig. S4
